# Efficacy of Intraarticular Vancomycin in Preventing Infection in Patients Undergoing Hip Hemiarthroplasty

**DOI:** 10.31661/gmj.v13i.3382

**Published:** 2024-10-18

**Authors:** Payam Mohammadhoseini, Seyed Mohammad Mohammadi, Amir Aghaei Aghdam, Mohsen Asgari

**Affiliations:** ^1^ Department of Orthopedics and Traumatology, Faculty of Medicine, Ahvaz Jundishapur University of Medical Sciences, Ahvaz, Iran

**Keywords:** Hip Hemiarthroplasty, Vancomycin, Infection, Intra-Articular

## Abstract

Background: Hip fractures are among the top ten causes of disability in adults worldwide. Patients with hip fracture are at significant risk of mortality and morbidity and reduced quality of life. The use of intra-wound vancomycin has been reported to be effective in reducing the incidence of infection in orthopedic surgeries. This study was conducted with the aim of investigating the effect of intra-articular vancomycin in preventing infection in patients undergoing hip hemiarthroplasty. Materials and Methods: This double-blind controlled clinical trial study was conducted on 48 patients with femoral neck fracture candidates for hip hemiarthroplasty hemiarthroplasty in Orthopedic clinic of Golestan and Imam Khomeini Hospital, Ahvaz, Iran between June and November 2023. Eligible patients were divided into two equal groups. The intervention group received 1gram vancomycin intra-articularly during the operation before closure of fascia, and the control group did not receive vancomycin. The patients were followed up for 6 months after the operation, and the rate of superficial infection, periprosthetic joint infection (PJI) and wound complications were compared in two groups. The obtained data were statistically analyzed with IBM SPSS Statistics 21.0 for Windows. Results: The vancomycin group and the control group had no significant difference in the incidence of overall infection. The PJI in vancomycin and control groups were 4.16% and 8.33%, respectively. This difference was not statistically considerable (P=0.55). The results showed the incidence of superficial estimated 8.33% in vancomycin group and 4.16% in control group with no considerable difference in infection (P=0.52). Moreover, there was no meaningful difference in side effects between the two groups (P=0.63). There was no significant difference in wound complications between the two groups (P=0.3). After the intervention, it was found that the ESR value in the control group and vancomycin group was 32.79±9.94, 31.83±9.78 mm/hr, respectively (P=0.73). Conclusion: Intra-articular injection of 1gram of vancomycin suspension did not reduce the overall, superficial and deep infection after surgery. It is suggested that more clinical trial studies with higher sample size be conducted in order to determine the effect of intra-articular vancomycin in preventing infection in patients undergoing hip hemiarthroplasty.

## Introduction

Hip fracture is one of the most common injuries, especially in the elderly, which is associated with high mortality and morbidity [1][2]. Currently, hip hemiarthroplasty is the standard method for the treatment of fractures with displacement of the femoral neck [3]. This procedure involves implanting a prosthesis for early recovery of mobility. Hip hemiarthroplasty relieves pain, improves long-term joint function, and increases the patient’s quality of life [3]. Surgical site infection including surface infections and periprosthetic joint infection (PJI) is one of the most common and serious complications after hip hemiarthroplasty [5]. The incidence of PJI in hemiarthroplasty after femoral neck fracture ranges from 2 to 17% [4][6].

PJI following joint arthroplasty can cause bone loss and significant damage to soft tissue structures [7][8], and as a result, it has a negative effect on the patient’s daily functioning and quality of life. The occurrence of PJI is associated with increased risk of mortality, increased length of hospital stay, and increased medical costs [9][10]. Several prophylaxis methods are performed before, during and after the operation to reduce the risk of postoperative infections [11]. Intravenous antibiotic administration of cephalosporin is usually used before surgery to prevent the risk of PJI [11][12], but culture of infections isolated from the wound in most cases has shown that the bacteria causing the infection are resistant to cephalosporin [13][14].

The two main bacteria causing deep infections are methicillin-resistant Staphylococcus aureus (MRSA) and coagulase-negative Staphylococcus. Therefore, the use of effective local antibiotics against these bacteria, such as vancomycin, can be useful [15].

Vancomycin is a glycopeptide antibiotic that has an antibacterial effect by inhibiting the synthesis of the cell wall of gram-positive bacteria [5][16]. The use of intra-wound vancomycin has been shown to be effective in reducing the incidence of infection in spine surgeries [17]. Although topical vancomycin is used to reduce the risk of infection in patients undergoing primary hip and knee arthroplasty. But currently, despite the promising initial results, there are significant debates and disagreements regarding the effectiveness of topical vancomycin in patients undergoing hip arthroplasty [2][18][19]. Therefore, the present study was conducted with the aim of investigating the effect of intra-articular vancomycin in preventing infection in patients undergoing hip hemiarthroplasty.

## Materials and Methods

**Figure-1:**
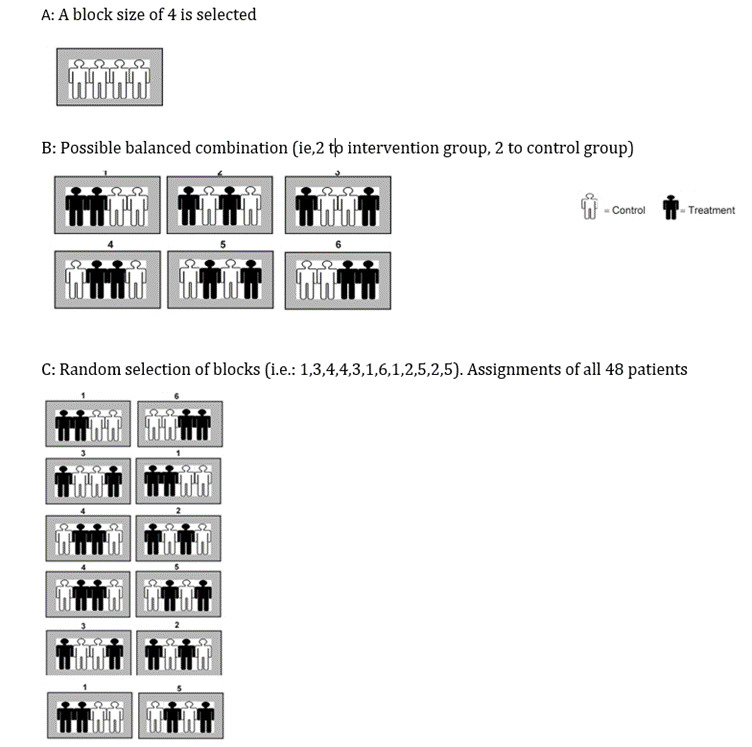
Figure 1. Block randomization procedure

Participant and study design

In this double-blind randomized clinical trial study 48 patients with femoral neck fractures who were candidates for hip hemiarthroplasty in Orthopedic clinic of Golestan and Imam Khomeini Hospital, Ahvaz, Iran between June and November 2023 were enrolled. The required sample size calculated based on the formula for the comparison of two independent means in literatures [20]. The sample size takes into account the required significance level and power of the test. Inclusion criteria were patients over 18 years of age, candidates for primary hip hemiarthroplasty and patient consent to participate. Patients with active, local or systemic infections, patients with osteoarthritis caused by infection, patients undergoing revision surgery and patients with known allergy to vancomycin were excluded from the study. At first, basic characteristics of patients including age, gender, underlying disease, cause of fracture and American Society of Anesthesiologists (ASA) score were collected. The eligible subjects were allocated in two groups of 24 patients using a four-block randomization method. The design of trial was parallel. One group was administered 1gram vancomycin intra-articularly (intervention group: I), and the other group did not receive vancomycin (control group: C). The methodology of block randomization is devised to allocate participants randomly into groups in order to achieve uniform sample sizes. The allocation ratio was 1:1. This approach is employed to guarantee an equitable distribution of sample size among groups throughout the duration of the study. Blocks consist of compact and equitable groupings with pre-established assignments, thereby maintaining a consistent number of participants in each group at all instances. For a clinical trial involving 48 participants divided into control and intervention groups, a randomized block procedure would be implemented as follows: Firstly, a block size of 4 is selected. Secondly, all possible balanced combinations with 2 subjects each for control (C) and intervention (I) groups are computed, resulting in 6 combinations (IICC, ICIC, ICCI, CIIC, CICI, CCII). Lastly, random selection of blocks is conducted to assign all 48 participants. By following this procedure, both the control and treatment groups will have 24 participants each (Figure-1). The random allocation was done by the supervisor.

Intra articular vancomycin was used before closing the wound. For patients in both groups, 30 minutes before skin incision, a dose of prophylactic antibiotic was administered intravenously (2gram cefazolin, and clindamycin in case of allergy). Except for intraoperative vancomycin, the pre-, intra- and post-operative treatment measures, other infection prevention protocols, as well as the surgical technique and the type of prosthesis used were similar for the two groups. All operations were performed by orthopedic surgeon under the identical surgery setting. Moreover, neither the participants nor the evaluator were unaware of the designed treatment groups. Patients did not know what they are getting, just they know they were participating in a research study (in aspect of ethical issue). The evaluator did not know which was the intervention and placebo (it was coded as A and B).

Evaluation of outcomes

The patients were followed up for 6 months after the surgery, and the rate of superficial and deep wound complications, erythrocyte sedimentation rate (ESR), and c-reactive protein (CRP) levels were evaluated and recorded in two groups. The incidence of joint infection around the prosthesis was investigated as the primary objective. Infection was diagnosed based on the Musculoskeletal Infection Society (MSIS) criteria [2]. Superficial infection was defined as surgical site infections that were treated with oral antibiotics and did not require further intervention [21]. The diagnosis of surgical site infection was based on four criteria: erythema and/or warmth, and/or itching, and/or increased local pain in the surgical wound site [22]. All cases of superficial or deep infection were treated according to standard protocols.

Ethical considerations

The ethical committee of Jundishapur University of Medical Sciences of Ahvaz has approved this study (IR. AJUMS. REC.1402.160), and this trial has been registered in the Iranian clinical trial system (IRCT20230703058652N1).

Statistical analysis

Statistical analysis was performed by SPSS software Version 22 (IBM, Chicago, USA). The quantitative and qualitative variables were indicated as mean±SD and number (percentage), respectively. Kolmogorov-Smirnov and, Shapiro-Wilk tests were used to test for the distribution. The incidence of joint infection around the prosthesis as the primary objective was evaluated via Chi-square test and the related Phi (φ) effect size is use for the chi-squared test. Comparison of inflammatory factors as the secondary outcome was done via the independent sample t-test and the related Cohen’s d effect size was estimated for comparing two groups. P-value<0.05 was considered statistically significant. The obtained data were statistically analyzed with IBM SPSS Statistics.

## Results

**Figure-2:**
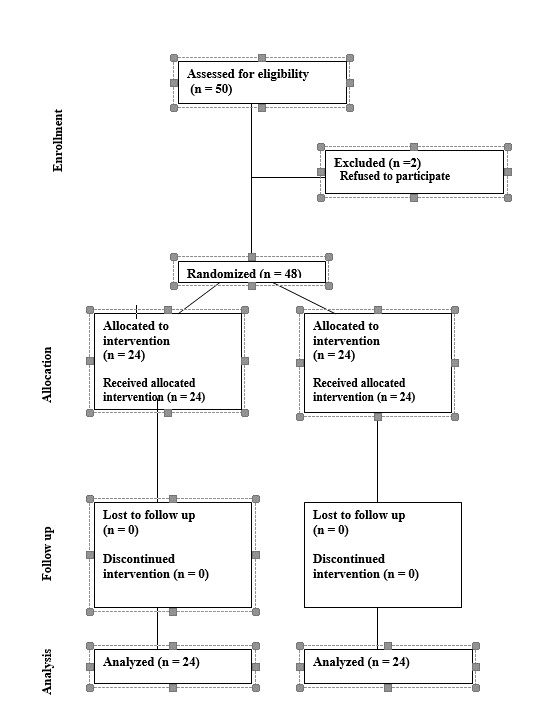
Figure 2. The CONSORT diagram showing the flow of participants through each stage of a randomized trial.

In this study, 50 patients were assessed for eligibility. Two cases were excluded since they refused to participate in a research study. Randomization were done for 48 patients and 24 patients allocated to each group separately. No one lost to follow up and discontinued the intervention during the study. All the 24 patients were included in the analysis. The Consort diagram is provided in Figure-2 and show these statements.

The mean ages of control and vancomycin groups were 74.04 ± 3.53 and 71.96 ± 5.32, respectively. The sex was distributed equally (14 females and 10 males in vancomycin and control group). The vancomycin and control groups did not have a significant difference in terms of presence of total infection after the intervention (P=0.98). The PJI in control and vancomycin groups were 8.33% and 4.16%, respectively. This difference was not statistically considerable (P=0.55). Superficial wound infection in the vancomycin group (8.33%) was higher than the control group (4.16%), but this difference was not significant (P=0.52). More details are provided in Table-1.

The Phi (φ) effect size estimated 0.01 for total infection, 0.001 for superficial wound infection and the 0.13 for periprosthetic joint infection. These findings show there is limited to no practical significance of the finding that the experimental intervention was more successful than the control intervention (Table-2).

The mean primary ESR levels in the control and vancomycin groups were 29.67±8.25 and 29.38±9.51 mm/hr, respectively (P=0.91). After the intervention, it was found that the ESR value in the control group and vancomycin group was 32.79±9.94, 31.83±9.78 mm/hr, respectively (P=0.73). There was no remarkable difference in the mean primary CRP levels in the control and intervention groups (P=0.96). Moreover, after the intervention, CRP levels did not differ between the two groups (P=0.35).

According to the Cohen’s d effect size, which are small values, there is limited to no practical significance of the finding that the experimental intervention was more successful than the control intervention.

In our study, there was no meaningful difference in side effects between the two groups (P=0.63). In addition, there was no significant difference in wound complications between the two groups (P=0.3).

## Discussion

**Table:** Table1. Demographic and clinical characteristics of the studied patients

Variable		Vancomycin group(n=24)	Control group(n=24)
Age (year)		71.96±5.32	74.04±3.53
Sex, n (%)	Female	14 (58.4)	14 (58.4)
	Male	10 (41.6)	10 (41.6)
Presence of total infection, n (%)		3 (12.5)	3 (12.5)
PJI, n (%)		1 (4.16)	2 (8.33)
Superficial wound infection, n (%)		2 (8.33)	1 (4.16)

**PJI:** periprosthetic joint infection

**Table:** Table2. Inflammatory factors in control and vancomycin groups before and after intervention

Variable	Vancomycin group(n=24)	Control group(n=24)	Cohen’s d effect size	P-value
Primary ESR (mm/hr)	29.38±9.51	29.67±8.25	0.01	0.91
ESR after intervention (mm/hr)	31.83±9.78	32.79±9.94	0.09	0.73
Primary CRP (mg/L)	17.08±5.06	17.00±7.15	0.01	0.96
CRP after intervention (mg/L)	15.04±5.72	17.17±9.46	0.27	0.35

**ESR:** erythrocyte sedimentation rate,**CRP:** C-reactive protein

Our findings showed the vancomycin group and the control group had no significant difference in the incidence of overall infection. The PJI in vancomycin and control groups were 4.16% and 8.33%, respectively. This difference was not statistically considerable (P=0.55). Some studies have indicated that the administration of intra-articular antibiotics is associated with a reduction in the rate of infection in total joint arthroplasty and other types of surgery with low risks of complications [23].

PJI is one of the most challenging conditions after total joint arthroplasty (TJA) [24]. Among all the strategies developed to prevent PJI, prophylactic antibiotics are still one of the most important methods. Recent research has shown that the incidence of PJI caused by methicillin-resistant S. aureus is increasing over time [25]. Vancomycin is recommended as an alternative antibiotic for high-risk patients. However, the safety of intravenous vancomycin is a concern because vancomycin toxicity may cause acute renal failure, ototoxicity, and anaphylaxis [26].

Due to the promising results of intra-articular injection of vancomycin in spine surgery and trauma, the interest in local administration of vancomycin during primary arthroplasty has increased in recent years. Several studies have investigated the effect of intralesional vancomycin on the prevention of PJI. However, consensus has not been reached and the topical use of vancomycin in primary arthroplasty is not accepted by most surgeons [27].

Matziolis et al., in study with a retrospective review of 8945 primary TJA, reported that the administration of 1 gram of intra-articular vancomycin powder significantly reduced the overall rate of PJI to 0.4%, and no local wound complications were observed [2].

Burns et al. reported the safety of a 1gram dose of intra-articular vancomycin in a series of primary hip and knee replacements [20]. Also, the study of Xu et al. founded a significant reduction in the level of PJI after injecting 0.5 grams of vancomycin powder into the joint cavity without increasing wound complications [28]. While, in our study, there was no significant difference between the vancomycin and control groups, PJI and superficial infection. It is possible that the small sample size of our study may be the reason for the difference with the study of Xu et al. The findings of Hanada et al.’s study on 166 patients undergoing primary total and unicompartmental knee arthroplasties demonstrated that the administration of intra-articular vancomycin does not reduce the incidence of PJI, in which 7 cases (7.6%) and 5 patients (4.5%) in the control and vancomycin groups had PJI [29]. The findings of Hanada’s study were in line with our results. Similar to our study, a systematic review of 3371 patients by Wong et al. did not indicate a significant decreasing in PJI in patients receiving vancomycin (0.19%) compared to the control group (0.58%) [30].

Contrary to our results, Dial et al. showed that vancomycin significantly reduced the risk of deep wound site infections from 5.5% to 7%, while the rate of sterile wound complication was not different in vancomycin group and control group. As a result, intra-wound vancomycin reduces the incidence of PJI and is associated with increased complications of wound sterility compared to control [20]. It is possible that the difference in the sample size is the reason for this discrepancy.

In the study by Xu et al., there were no serious side effects associated with topical vancomycin [28]. Similar to our study, side effects were not significantly different in two groups. Our findings showed that was no significant difference in wound complications between the two groups, while Xu et al. reported that the incidence of local wound complications was higher in the vancomycin group in patients undergoing hip and knee arthroplasty [15].

The main limitation of the study is that due to the monocentric design, the number of cases included is small, which potentially increases the chance of confounding variables. The strength of this investigation is the prospective randomized study design.

## Conclusion

Intra-articular injection of 1 gram of vancomycin suspension did not reduce the incidence of total, superficial and periprosthetic joint infection after surgery compared to the control group. More prospective multicenter, adequately powered trials with higher sample size demonstrating a clear reduction in the risk of infection are suggested before recommending the widespread use of intra-articular vancomycin.

More multicenter clinical trial studies with higher sample size are suggested to investigate the effect of intra-articular vancomycin in preventing infection in patients undergoing hip hemiarthroplasty.

## Conflict of Interest

The authors declare that they have no competing interests.
